# Introduction to ‘The Epitranscriptome’

**DOI:** 10.1039/d4cb90006e

**Published:** 2024-03-06

**Authors:** Ralph E. Kleiner, Claudia Höbartner, Guifang Jia

**Affiliations:** a Department of Chemistry, Princeton University Princeton 08544 NJ USA; b Institute of Organic Chemistry, University of Würzburg Am Hubland Würzburg 97074 Germany; c College of Chemistry and Molecular Engineering, Peking University Beijing 100871 China guifangjia@pku.edu.cn

## Abstract

Ralph Kleiner (Princeton University, USA), Claudia Höbartner (University of Würzburg, Germany) and Guifang Jia (Peking University, China) introduce the themed collection on ‘The Epitranscriptome’.
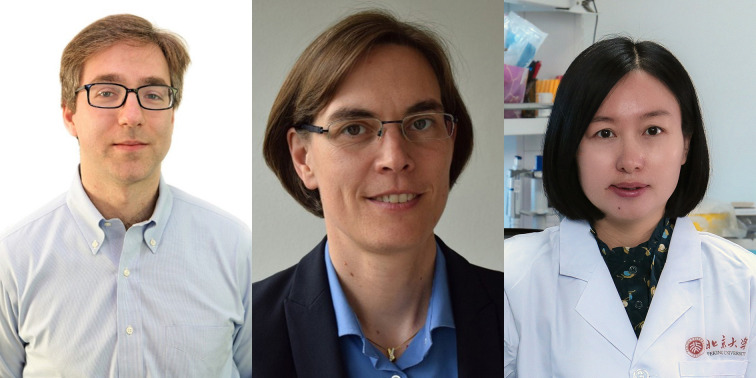

This themed collection presents articles in the field of epitranscriptomics, delving into the exploration of non-canonical ribonucleotides in biology. Modified RNA nucleotides play important roles in biological processes and can be exploited for therapeutic benefit. Recent years have seen an explosion of studies aimed at deciphering the chemical and biological complexity of the RNA epitranscriptomic code and harnessing RNA modifications and RNA-modifying enzymes for programmable gene-expression modulation. In particular, researchers have directed their efforts towards developing new methods for the comprehensive characterization of RNA modifications, primarily relying on advanced mass spectrometry and nucleic-acid sequencing technologies. Investigations have extended to understanding the regulation of RNA modifications by cellular enzymes, elucidating the effects of modified nucleotides on the biochemical properties of RNA molecules, and discerning the function of RNA modification pathways in higher-order biological processes. Finally, the therapeutic potential of modified ribonucleotides is a focal point of interest in the field. This encompasses applications in mRNA therapeutics, exemplified by the success of COVID-19 mRNA vaccines, as well as approaches for the targeted installation of RNA modifications through chemoenzymatic strategies.

Precise mapping and quantification of RNA modifications at single-nucleotide resolution across the transcriptome is one of the grand challenges in the field of epitranscriptomics. In this collection, Burrows and co-workers apply nanopore direct RNA sequencing in conjunction with bisulfite chemistry to sequence pseudouridine and 5-methylcytidine sites in *E. coli* transcripts. This methodology, termed “chemically assisted nanopore sequencing”, offers distinct advantages over nanopore sequencing of native RNA modifications (https://doi.org/10.1039/D3CB00081H).

Liquid chromatography-mass spectrometry stands out as a powerful tool to quantify the levels of various RNA modifications. The Kaiser lab presents a methodological improvement for their stable isotope-labeling coupled nucleic acid mass spectrometry method, NAIL-MS. They employ the transcription inhibitor actinomycin D to increase the temporal resolution of their approach, thereby enabling the dissection of mechanisms underlying nucleotide dynamics and turnover in diverse RNA species (https://doi.org/10.1039/D2CB00243D).

The levels of RNA modifications are dynamically regulated by cellular ‘writer’ and ‘eraser’ enzymes. Cahová and co-workers investigate the RNA decapping activity of Nudix enzymes from *Arabidopsis thaliana* on non-canonically capped RNA species. Among the enzymes tested, they identify decapping activity on NAD/H-capped RNA and Ap_4_A-capped RNA with distinct substrate preferences exhibited by individual enzymes. Their findings implicate these proteins in the regulation of non-canonically capped RNA transcripts (https://doi.org/10.1039/D2CB00213B).

The epitranscriptomic modification inosine (I) possesses a remarkable capability to recode translation. Reprogramming the enzymes responsible for installing inosine, known as ADARs, is emerging as a promising strategy to correct disease-causing mutations present in protein-coding sequences. One approach for ADAR reprogramming involves the use of synthetic guide RNAs that target ADAR enzyme activity to specific sites in the transcriptome. In this contribution from the Beal lab, they investigate the impact of incorporating nucleoside analogs into ADAR guide RNA strands. They find that synthetic nucleoside analogs, including 2′-deoxynebularine and locked nucleic acid (LNA), can tailor the properties of guide-strand-directed ADAR activation, facilitating more efficient and specific inosine formation (https://doi.org/10.1039/D2CB00165A).

Taken together, we hope that readers will find this small sampling of epitranscriptomic research, showcasing recent directions in the field, to be a stimulating and thought-provoking entry point for further reading and study.

## Supplementary Material

